# Association Between Thyroid Hormone Levels and Disease Prognosis in Guillain–Barré Syndrome: A Retrospective Study

**DOI:** 10.1002/hsr2.70818

**Published:** 2025-05-15

**Authors:** Yangrongzhuo Huang, Yuhan Li, Hailing Zhou, Juan Tang

**Affiliations:** ^1^ Department of Neurology The First Affiliated Hospital of Shihezi University Shihezi China

**Keywords:** clinical characteristics, free thyroxine, Guillain–Barré syndrome, inflammatory markers, prognosis, thyroid hormone levels, thyroxine

## Abstract

**Introduction/Aims:**

Guillain–Barré syndrome (GBS) is an immune‐mediated neuropathy characterized by progressive sensory and motor dysfunction, often accompanied by abnormal inflammatory markers and thyroid hormone levels. However, the underlying mechanisms and their impact on prognosis remain incompletely understood. This study aimed to investigate the correlation between thyroid hormone levels and prognosis in GBS, analyze the association between thyroid hormone levels and inflammatory markers, and further explore potential mechanisms and clinical implications.

**Methods:**

We retrospectively analyzed clinical data from 182 GBS patients admitted to the First Affiliated Hospital of Shihezi University between December 2019 and April 2024. Data included thyroid hormone levels and inflammatory markers (e.g., neutrophils, leukocytes). Functional status was assessed using the Hughes Functional Grading Scale (HFGS) within 3 months post‐discharge. Patients were stratified into two groups: HFGS score < 3 (good prognosis group, *n* = 66) and ≥ 3 (poor prognosis group, *n* = 116). Logistic regression identified prognostic risk factors, Receiver operating characteristic (ROC) curves determined cut‐off values for FT4 and T4, and correlation analyses evaluated relationships between thyroid hormone levels and inflammatory markers.

**Results:**

Reduced FT4 and T4 levels were significantly associated with poor prognosis in GBS patients (*p* < 0.05). Spearman correlation analysis demonstrated significant associations between thyroid hormones and inflammatory markers. FT3 exhibited negative correlations with erythrocyte sedimentation rate (ESR) (*r* = −0.342, *p* < 0.01) and neutrophil count (*r* = −0.205, *p* < 0.05), whereas FT4 was positively correlated with NLR (*r* = 0.219, *p* < 0.05) and T4 levels (*r* = 0.506, *p* < 0.01). T3 was inversely associated with neutrophil count (*r* = −0.220, *p* < 0.05). Among inflammatory markers, PLR showed a strong positive correlation with NLR (*r* = 0.671, *p* < 0.01), and WBC count was highly correlated with neutrophil count (*r* = 0.889, *p* < 0.01). These findings suggest a potential interplay between thyroid hormone regulation and systemic inflammatory responses.

**Conclusion:**

This study suggests that low FT4 and T4 levels are independent risk factors for poor prognosis in GBS patients, with thyroid hormone levels exhibiting certain associations with inflammatory markers.

## 1 Introduction

GBS is an infection‐associated, immune‐mediated peripheral neuropathy characterized by acute or chronic progressive sensory and motor dysfunction, often accompanied by autonomic nervous system abnormalities. Although therapeutic agents such as corticosteroids, plasma exchange (PE), and intravenous immunoglobulin (IVIG) demonstrate significant efficacy, the accurate early prediction of prognosis and proactive intervention in relevant factors are critical for management and improving patients' quality of life.

In recent years, thyroid hormones have received considerable attention in neurological disorders. These hormones not only directly influence the central nervous system by crossing the blood–brain barrier, but also indirectly modulate the pathogenesis and progression of several neurological disorders through their involvement in immune regulation and inflammatory responses. The thyroid hormone system comprises triiodothyronine (T3), thyroxine (T4), and their free forms (FT3 and FT4), which are synthesized and secreted by the thyroid gland. Their release is regulated by thyroid‐stimulating hormone (TSH), a glycoprotein secreted by the anterior pituitary gland. The circulating free fractions (FT3 and FT4) directly reflect the functional status of the thyroid gland, while TSH serves as a sensitive biomarker of hypothalamic‐pituitary‐thyroid axis activity.

Emerging evidence indicates that abnormal thyroid hormone levels in GBS patients are strongly associated with disease severity and unfavorable prognosis [1]. However, studies investigating the mechanistic role of thyroid hormones in GBS progression and their clinical implications remain limited. This study aims to (1) explore the correlation between thyroid hormone profiles and GBS prognosis, and (2) evaluate their potential as prognostic biomarkers, thereby providing novel theoretical foundations for clinical decision‐making and therapeutic strategies.

## 2 Subjects and Methods

### 2.1 Subjects

This study adhered to the Strengthening the Reporting of Observational Studies in Epidemiology (STROBE) guidelines. The research protocol was approved by the Ethics Committee of the Affiliated Hospital of Shihezi University (approval KJ2024‐191‐01). As all clinical data utilized in this study were fully anonymized with no identifiable personal information, the requirement for informed consent was waived in accordance with national regulations and institutional guidelines.

This study enrolled 182 patients diagnosed with GBS at the First Affiliated Hospital of Shihezi University between December 2019 and April 2024, all of whom met predefined inclusion and exclusion criteria. Data were extracted from the hospital's electronic medical record (EMR) system, encompassing clinical characteristics, demographic information, laboratory findings, and results from telephone follow‐ups conducted 3 months post‐discharge. To ensure data accuracy, all records were independently reviewed by two professionally trained researchers, with discrepancies resolved by consensus discussion or adjudication by a senior investigator when necessary.

Inclusion criteria:

1. Patients meeting internationally recognized diagnostic criteria for GBS and its variants [2];
2. Age < 90 years to minimize age‐related physiological variations, ensure sample homogeneity, and increase generalizability of findings.

Exclusion criteria:

1. Patients with diagnoses of thyroid disorders (treated or untreated), to avoid overlapping manifestations between thyroid dysfunction and GBS symptoms;
2. Patients with other neurological diseases (e.g., subarachnoid hemorrhage, encephalitis) or their sequelae to ensure exclusive inclusion of GBS‐specific clinical features;
3. History of poliomyelitis, periodic paralysis, dermatomyositis, myasthenia gravis, acute rhabdomyolysis, or Lyme disease;
4. History of infection with *Corynebacterium diphtheriae* (diphtheria);
5. Moderate to severe peripheral neuropathy caused by heavy metals, medications, botulinum toxin, or other toxins to exclude non‐GBS aetiologies;
6. Previous exposure to drugs known to induce neuropathy or mimic GBS symptoms;
7. Hysterical paralysis secondary to psychiatric illness;
8. Psychiatric history that may compromise the reliability of clinical assessments or diagnosis.

### 2.2 Methods

#### 2.2.1 Patient Information and Data Collection

Demographic and clinical data were collected for all enrolled patients, including age, sex, and season of onset. Clinical symptoms were categorized as motor deficits (MD), sensory disturbances (SD), and autonomic dysfunction (AD). Laboratory investigations comprised: cerebrospinal fluid (CSF) antibody testing; imaging studies (CT or radiography); electromyography (EMG) to assess nerve conduction abnormalities. Serological analyses included: thyroid hormone profiles: TSH, T3, FT3, T4, and FT4; complete blood counts: neutrophil, leukocyte, and lymphocyte count, with derived ratios; Neutrophil to lymphocyte ratio (NLR) = neutrophil count/lymphocyte count; Platelet to lymphocyte ratio (PLR) = platelet count/lymphocyte count. Therapeutic interventions were documented as corticosteroid therapy (CS) and IVIG therapy.

#### 2.2.2 Assessing the Severity of Neurological Dysfunction

The HFGS [3] was used to evaluate patients' follow‐up conditions post‐discharge. A score of 1–2 indicated a good group, while scores of 3–6 indicated a poor prognosis group.

### 2.3 Statistical Analysis

All statistical analyses and graphical representations were performed using SPSS version 26.0 (IBM Corp, Armonk, NY, USA) and Origin 2024b (Origin Lab Corporation, Northampton, MA, USA). Normally distributed continuous variables were expressed as mean ± standard deviation (x̄ ± SD) and compared using independent samples *t*‐tests. Normality was assessed via the Kolmogorov–Smirnov test. Non‐normally distributed data were presented as median with interquartile range [M(Q1–Q3)] and analyzed using non‐parametric Mann–Whitney *U* tests. Categorical variables were compared via Pearson's *χ* ^2^ or Fisher's exact tests, as appropriate. Multivariate logistic regression identified risk factors for poor prognosis in GBS patients. ROC curve analysis determined optimal cut‐off values for predictive biomarkers. Spearman's rank correlation (correlation coefficient *r* ranging from −1 to +1) evaluated associations between inflammatory markers and thyroid hormone levels. A two‐tailed *p*‐value < 0.05 was considered statistically significant.

## 3 Result

### 3.1 Basic Information of Patients With GBS

A total of 182 GBS patients were included in this study, with 148 males (81.3%) and 34 females (18.7%), yielding a male‐to‐female ratio of 4.4:1—higher than previously reported ratios in the literature [4]. The mean age was 54.46 ± 16.20 years (range: 48–62 years).

The seasonal distribution of onset was 38 cases (20.9%) in spring, 66 (36.3%) in summer, 34 (18.7%) in autumn, and 40 (22.0%) in winter. Regarding preceding infections, 80 patients (44.0%) had no identifiable infectious triggers, while antecedent events included SARS‐CoV‐2 positivity in 28 (15.4%), diarrheal episodes in 10 (5.5%), and trauma‐associated wound infections in 2 (1.1%).

The standard hospitalization duration was 14–28 days. Twenty‐two patients (12.1%) required prolonged hospitalization (> 28 days), whereas 160 (87.9%) were discharged within 28 days. Based on the HFGS at 3‐month follow‐up, 116 patients (63.7%) were classified into the poor prognosis group, and 66 (36.3%) into the favorable prognosis group.

### 3.2 Clinical Symptoms, Additional Tests, and Treatment Options

Motor and sensory deficits were nearly universal, with 178 patients (97.8%) exhibiting limb muscle weakness and 178 (97.8%) reporting sensory disturbances (numbness or pain). Cranial nerve involvement was observed in 112 patients (61.5%) and manifested as ophthalmoplegia, facial paralysis, dysphagia, choking episodes, or dysarthria. Autonomic dysfunction—present in all patients (100%)—included cardiac arrhythmias, abnormal sweating, blood pressure fluctuations, gastrointestinal symptoms (bloating, nausea, constipation, or diarrhea), and thermoregulatory disturbances. Additionally, 60 patients (33.0%) experienced urinary/bowel dysfunction. Respiratory compromise necessitating mechanical support occurred in 70 patients (38.5%), with 38 (20.9%) requiring tracheostomy and 42 (23.1%) undergoing endotracheal intubation.

Laboratory and diagnostic investigations revealed the following: CSF antibody testing was performed in 44 patients (24.2%), with negative results in 5 cases (11.4%). Positive findings included anti‐GD1b antibodies in 26 patients (14.3% of the total cohort) and anti‐GM1 antibodies in 24 patients (13.2%). Imaging studies demonstrated pulmonary inflammatory signs in 92 patients (50.5%) via chest CT or radiography. EMG, performed in 168 patients (92.3%), identified normal nerve conduction velocities and amplitudes in 2 patients (1.2% of the cohort tested), isolated demyelinating involvement in 14 (8.3%), primary axonal damage in 20 (11.9%), and mixed axonal‐demyelinating pathology in 132 (78.6%).

### 3.3 Comparison Between Good and Poor Prognosis Groups

Based on the Hughes scores (GBS disability scores) assessed at 3–6 months post‐discharge, patients were categorized into a favorable prognosis group (score < 3, *n* = 66) and a poor prognosis group (score ≥ 3, *n* = 116). The analysis of baseline characteristics revealed that age, gender, season of onset, T4 levels, FT4 levels, as well as the presence of motor and sensory deficits, constituted significant prognostic factors influencing disease outcomes (detailed in Table 1).

**Table 1:** Comparison of baseline characteristics between good and poor prognosis groups.

	Good prognosis groups (*n* = 66)	Poor prognosis groups (*n* = 116)	*t/Z/Χ*²	*p*
Age	61.00 (59.00–66.00)	53.00 (44.00–59.00)	−4.353	< 0.001
Gender (male)	48 (72.73%)	100 (86.21%)	−2.237	0.025
Season			−3.576	< 0.001
Spring	17 (25.76%)	25 (21.55%)		
Summer	33 (50.00%)	33 (28.44%)		
Autumn	16 (24.24%)	18 (15.52%)		
Winter	—	40 (34.49%)		
Neutrophil	5.19 (3.21–6.83)	5.15 (3.16–7.23)	−0.076	0.939
Lymphocyte	1.80 (1.08–2.43)	1.80 (0.99–2.40)	−0.094	0.925
ESR	19.00 (11.75–19.00)	19.00 (14.00–19.00)	−1.380	0.167
NLR	2.65 (1.41–5.93)	2.79 (1.53–6.15)	−0.275	0.783
PLR	148.00 (106.46–228.54)	141.67 (103.01–239.69)	−0.199	0.842
TSH	2.54 (1.40–2.54)	2.54 (1.52–2.90)	−0.924	0.355
T3	1.52 (1.52–1.52)	1.52 (1.52–1.52)	−0.594	0.553
FT3	4.32 (3.91–4.54)	4.32 (3.95–4.56)	−0.390	0.696
T4	94.64 (85.64–99.14)	99.14 (99.14–113.23)	−6.042	< 0.001
FT4	17.72 (17.55–18.35)	18.74 (17.72–24.25)	−3.615	< 0.001
MD	62 (93.94%)	116 (100%)	−2.674	0.008
SD	62 (93.94%)	116 (100%)	−2.674	0.008
CS	54 (81.82%)	100 (86.21%)	0.787	0.431
IVIG	40 (60.61%)	60 (51.72%)	1.155	0.248

### 3.4 Logistic Regression Analysis of Prognostic Factors in GBS

**Figure 1:**
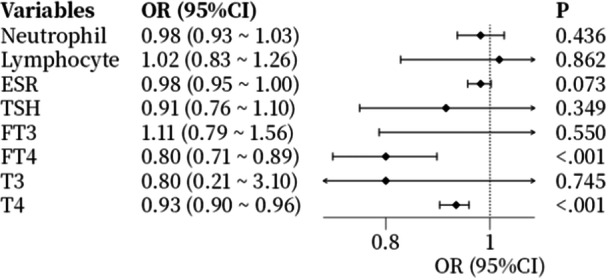
Forest plot of univariate logistic regression analysis for prognostic factors in GBS.

Univariate logistic regression analysis was performed to identify factors associated with prognosis (Figure 1). The results demonstrated that both T4 and FT4 levels were significant risk factors influencing outcomes in GBS patients.

Subsequently, a multivariate logistic regression model was constructed using T4 and FT4 (identified as statistically significant in univariate analysis) as independent variables, while adjusting for potential confounders including age and gender (Table 2). The analysis confirmed that T4 and FT4 remained independently associated with prognosis after adjustment.

**Table 2:** Multivariate logistic regression analysis of prognosis factors in GBS.

	B	SE	Wald	*p*	OR	95% CI
T4	−0.068	0.019	13.109	< 0.001	0.937	0.905–0.971
FT4	−0.133	0.064	4.366	0.037	0.875	0.772–0.992

**Figure 2:**
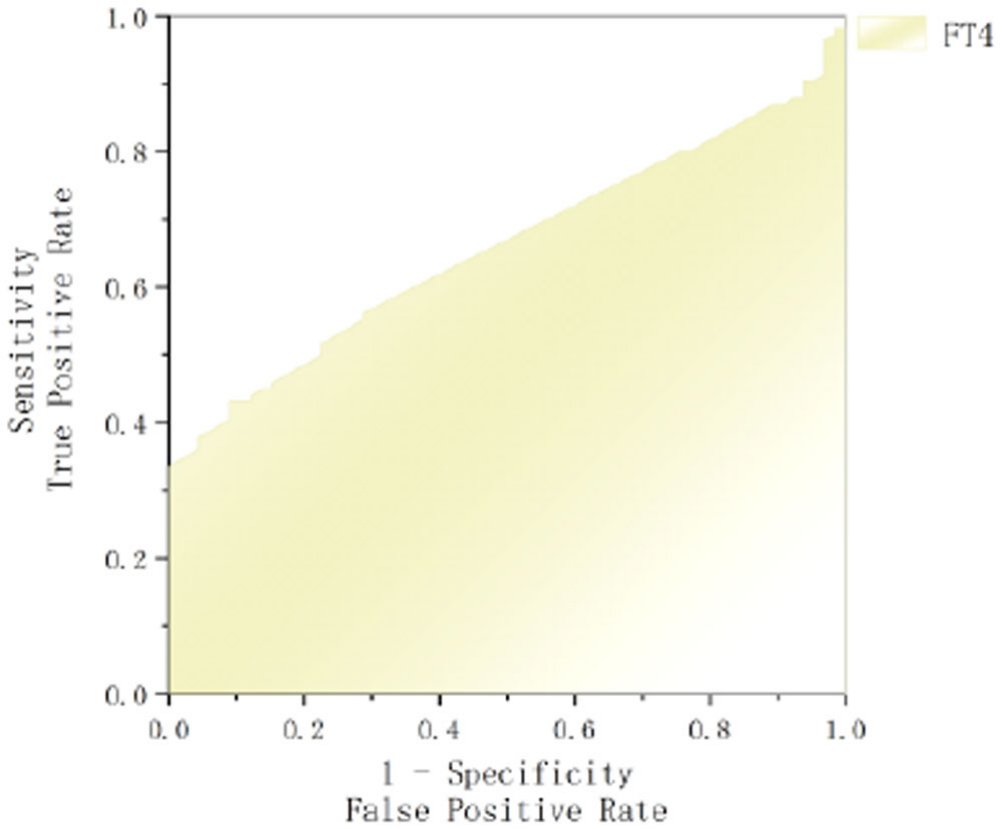
ROC curves comparing FT4 for predicting poor prognosis in GBS.

**Figure 3:**
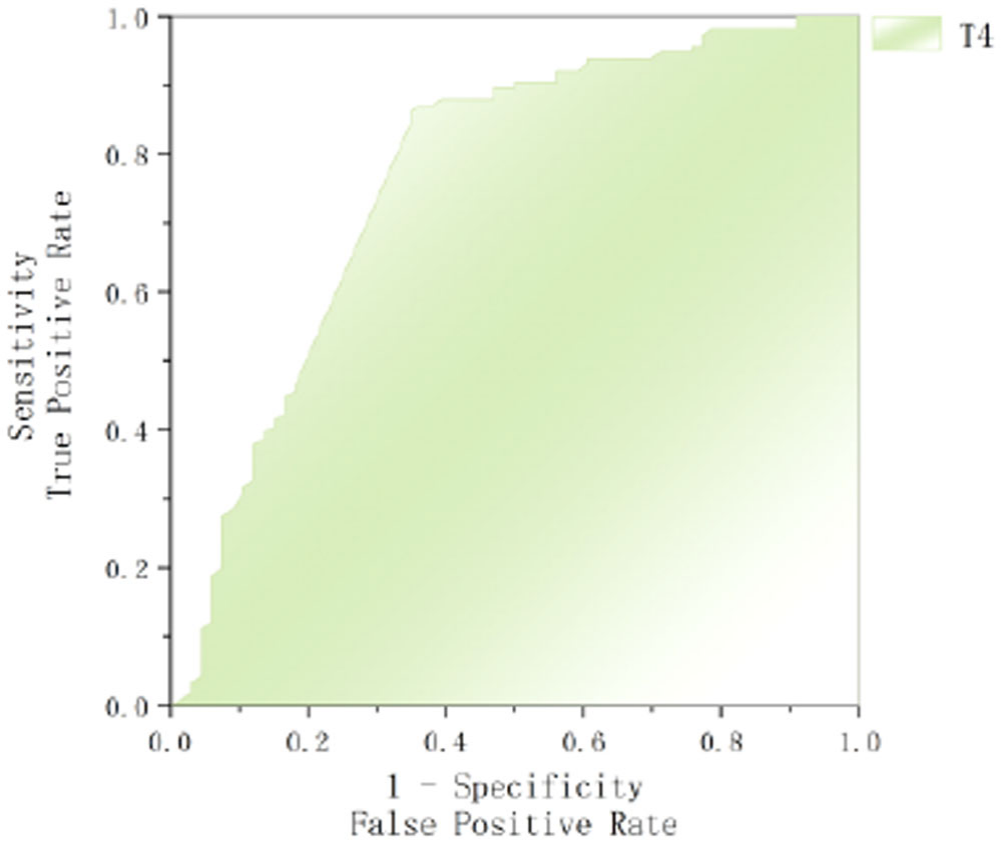
ROC curves comparing T4 for predicting poor prognosis in GBS.

To evaluate the predictive performance of FT4 and T4 for GBS prognosis, ROC curve analysis was conducted, with poor prognosis as the state variable. The area under the curve (AUC) for FT4 was 0.660 (95% CI: 0.583–0.736, *p* < 0.001), with an optimal cut‐off value of 20.49 pg/mL, yielding a sensitivity of 43.1% and specificity of 90.9%. For T4, the AUC was 0.766 (95% CI: 0.689–0.843, *p* < 0.001), with an optimal cut‐off value of 98.69 pg/mL, demonstrating a sensitivity of 86.2% and specificity of 65.2%. These findings indicate that lower levels of T4 and FT4 are associated with an increased risk of poor prognosis (Figures 2 and 3).

### 3.5 Correlation Analysis Between Thyroid Hormone Levels and Inflammatory Markers

Spearman correlation analysis was performed to investigate the relationships between thyroid hormone levels and inflammatory markers. The results demonstrated that: FT3 showed significant negative correlations with ESR (*r* = −0.342, *p* < 0.01) and neutrophil count (*r* = −0.205, *p* < 0.05); FT4 exhibited positive correlations with NLR (*r* = 0.219, *p* < 0.05) and T4 levels (*r* = 0.506, *p* < 0.01), highlighting a potential link between T4 and immune cell dynamics; T3 was negatively correlated with neutrophil count (*r* = −0.220, *p* < 0.05), further supporting the role of thyroid hormones in modulating inflammatory responses.

**Figure 4:**
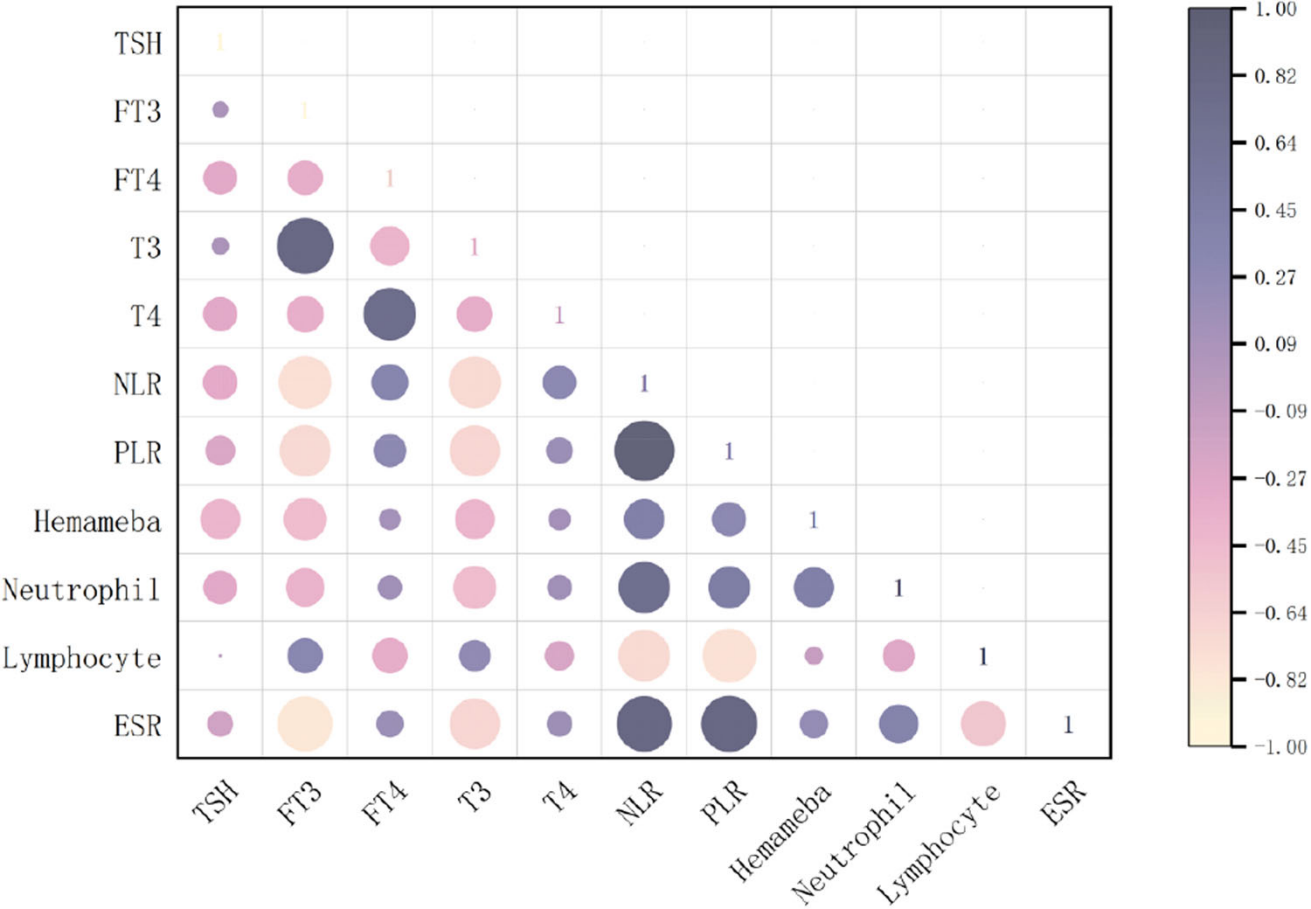
Correlation heatmap of thyroid hormones and inflammatory markers in GBS.

Among inflammatory markers: PLR positively correlated with NLR (*r* = 0.671, *p* < 0.01); White blood cell count strongly correlated with neutrophil count (*r* = 0.889, *p* < 0.01). These findings emphasize significant interactions between thyroid hormone profiles and systemic inflammatory indicators (Figure 4).

## 4 Discussion

GBS, an acute post‐infectious autoimmune polyradiculoneuropathy, demonstrates an incidence of approximately 0.9 per 100,000 person‐years in Chian with an age‐dependent increase. This study's median onset age of 51 years aligns with established epidemiological trends, while the observed male predominance (male‐to‐female ratio 4.4:1) significantly exceeds literature‐reported ratios (1.5:1) [4, 5], potentially reflecting regional epidemiological variations, healthcare access disparities, or single‐center selection bias. Additionally, we identified significant seasonal clustering during summer months [6], which may correlate with latitude‐specific winter suppression of antecedent pathogen activity under cold‐dry climatic conditions.

While IVIG and PE constitute standard first‐line therapies for GBS, our analysis revealed no significant difference in clinical outcomes between treatment modalities (*p* > 0.05). Potential explanations include: first, insufficient statistical power due to high IVIG usage (54.95%) and overlapping treatments. Second, limited therapeutic efficacy of current immunotherapies in altering disease course; and third, stronger prognostic influence of patient‐specific efficacy in heterogeneous GBS cohorts, underscoring the need for stratified treatment approaches.

This study systematically investigated the association between thyroid function parameters and GBS prognosis, revealing for the first time that FT4 levels < 20.49 pg/mL and T4 levels < 98.69 pg/mL are strongly predictive of poor clinical outcomes. Given that FT4 is the biologically active form of T4, its dysregulation—often observed in immune‐mediated conditions such as post‐infectious thyroiditis—may disrupt thyroid hormone homeostasis and impair systemic immune‐microenvironmental balance. These findings align with prior evidence: Wang et al. demonstrated that elevated TSH levels increases recurrence risk in GBS [7], while other studies associate low TSH and elevated FT4 with higher GBS incidence and severity [8]. Furthermore, in a cohort of 219 GBS patients, reduced low FT3 levels were linked to exacerbating oxidative stress and accelerated disease progression [9]. Collectively, these data suggest that thyroid dysfunction may serve as both a biomarker and a modulator of GBS severity, warranting further mechanistic investigation.

Thyroid hormones critically regulate both innate and adaptive immunity [1]. In GBS, immune activation, inflammatory cytokine release, and impaired antioxidant defense drive excessive reactive oxygen species (ROS) production [2, 3]. Elevated ROS levels can stimulate T‐cell activation and enhance Fas signaling, promoting apoptosis. Fas (CD95), a tumor necrosis factor receptor (TNF‐R) family member, initiates downstream cascades upon FasL binding and plays a key role in immune tolerance by eliminating autoreactive T‐cell [5]. Dysregulated Fas signaling may allow autoreactive lymphocytes to persist, exacerbating autoimmunity. Furthermore, oxidative stress can amplify Fas‐mediated apoptosis, potentially worsening GBS progression.

Thyroid hormones play a critical role in modulating both innate and adaptive immunity [10, 11]. In GBS, immune activation, inflammatory cytokine release, and impaired antioxidant defenses contribute to disease pathogenesis. Laman et al. proposed that the comorbidity of thyroid disorders and immune‐mediated neurological diseases may arise from shared immune dysregulation [12]. This associate is further supported by long‐standing evidence linking thyroid hormone levels to demyelinating diseases [13], as well as broader observations by Duyff et al. connecting thyroid dysfunction and various neurological conditions [14]. Clinical evidence, including case reports by Ahn et al., strengthens this potential link, documenting instances of GBS co‐occurring with thyroid storm and Hashimoto's thyroiditis [15]. Mechanistically, molecular mimicry may serve as a key trigger, wherein cross‐reactive antigens activate T cells, driving cytokine release and Fas‐mediated apoptosis. These processes disrupt oxidative stress homeostasis and promote ganglioside antibody production, which could simultaneously drive thyroid hormone abnormalities and GBS development. Thus, thyroid dysfunction may not merely coexist with GBS but could actively participate in its immunopathogenesis.

This study systematically investigates the correlation between thyroid hormone levels and inflammatory markers in GBS patients. While the results suggest a potential association, this relationship may be influenced by confounding factors such as limited sample size and concurrent medication use. Notably, NLR, PLR, leukocyte count, and neutrophil count demonstrated positive correlations with disease severity, while lymphocyte count demonstrated a negative correlation. These findings highlight the critical role of dysregulated immunoinflammatory pathways in GBS pathogenesis. Elevated NLR, PLR, and neutrophil are associated with heightened systemic inflammation and poorer prognoses, whereas lymphocytopenia may indicate compensatory immunosuppression. The dynamic interplay among these hematological indices suggests that the immune system exerts bidirectional control over inflammatory responses through complex cellular crosstalk during disease progression, ultimately influencing clinical trajectories.

In GBS patients, microbial infections initiate a complex immunopathological cascade through both direct and indirect mechanisms [6]. The resultant T‐cell activation drives the production pro‐inflammatory cytokines, including interleukin‐6 (IL‐6) and tumor necrosis factor‐alpha (TNF‐α), which subsequently elevate systemic inflammatory markers such as ESR and C‐reactive protein (CRP) levels [16, 17]. Concurrently, this inflammatory milieu promotes neutrophil recruitment while simultaneously inducing lymphocyte apoptosis and suppressing lymphopoiesis, manifesting as an elevated NLR. Importantly, emerging evidence suggests that immune cells may disrupt thyroid hormone homeostasis through cytokine‐mediated effects (particularly interferon signaling) and altered intercellular communication, resulting in characteristic endocrine perturbations including decreased TSH and elevated FT4/FT3 levels [18]. These pathophysiological interactions provide a mechanistic basis for the observed clinical correlations: the positive association between FT4 and NLR, the direct relationship between TSH and ESR, and the inverse correlation between FT3 and ESR, collectively highlighting the intricate crosstalk between neuroendocrine and immune systems in GBS pathogenesis.

Thyroid hormones exhibit a biphasic regulatory influence on neutrophil function. On one hand, they may enhance neutrophil activity by stimulating ROS production [19]; conversely, they can suppress neutrophil generation or function through interference with cell surface receptors, thereby attenuating ROS generation [20]. This dual regulatory mechanism aligns with the observed negative correlation between FT3 and neutrophil count in this study. Further supporting these findings, Wang et al. demonstrated that NLR and PLR serve as independent predictors of TSH and FT3 levels, while PLR independently associates with FT4 [21]. Notably, T4 levels may represent a clinically relevant biomarker for assessing disease severity in inflammatory conditions [21]. Collectively, these observations underscore a dynamic interplay between thyroid hormone homeostasis and systemic inflammation [22], mediated through multiple mechanisms including immune modulation, inflammatory cascades amplification, and metabolic reprogramming across physiological and pathological states.

The findings of this study demonstrate that reduced levels of T4 and FT4 are associated with an increased risk of adverse prognostic events, and highlighting a significant correlation between thyroid hormone levels and systemic inflammatory markers. These results underscore the clinical relevance of integrating thyroid function and inflammatory biomarker monitoring into patient management strategies to facilitate timely interventions. However, several limitations should be acknowledged. First, this study was conducted at a single center with a relatively small sample size, which limits the generalizability of the findings. Second, the retrospective design relied on database‐derived clinical data and telephonic follow‐ups, potentially introducing reporting biases in patient status assessments. Additionally, the short follow‐up duration restricted the evaluation of long‐term outcomes, and the absence of longitudinal tracking of thyroid hormones and inflammatory markers in enrolled patients may partially compromise the robustness of conclusions. Future multicenter, prospective studies with larger cohorts and extended follow‐up periods are warranted to validate these and further elucidate the underlying mechanisms.

In summary, this study establishes T4 and FT4 as novel prognostic biomarkers in GBS, offering clinically actionable evidence to refine prognostic assessments. Our finding underscores a potential pathophysiological interplay between thyroid hormone dysregulation and neuroinflammatory processes, advancing the mechanistic understanding of GBS progression. However, whether these effects are mediated via immune modulation, neuroendocrine pathways, or other biological cascades requires further investigation. Future research should prioritize elucidating thyroid‐immune crosstalk to guide precision therapies, alongside rigorous comparative studies of corticosteroids and IVIG efficacy across patient subgroups—particularly those with thyroid dysfunction—to optimize treatment protocols, mitigate complications, and improve long‐term outcomes.

## Author Contributions

**Yangrongzhuo Huang:** writing – review and editing and writing – original draft. **Yuhan Li:** data curation and investigation. **Hailing Zhou:** formal analysis and software. **Juan Tang:** methodology and supervision.

## Conflicts of Interest

The authors declare no conflicts of interest.

## Transparency Statement

The corresponding author Juan Tang, affirms that this manuscript is an honest, accurate, and transparent account of the study being reported; that no important aspects of the study have been omitted; and that any discrepancies from the study as planned (and, if relevant, registered) have been explained.

## Data Availability

The data that support the findings of this study are available from the corresponding author upon reasonable request.
